# Graphene-enhanced Raman scattering on single layer and bilayers of pristine and hydrogenated graphene

**DOI:** 10.1038/s41598-020-60857-y

**Published:** 2020-03-11

**Authors:** Václav Valeš, Karolina Drogowska-Horná, Valentino L. P. Guerra, Martin Kalbáč

**Affiliations:** 0000 0004 0633 9822 grid.425073.7 J. Heyrovský Institute of Physical Chemistry, ASCR, v.v.i., Dolejškova 3, 182 23 Praha, Czechia

**Keywords:** Materials science, Nanoscience and technology

## Abstract

Graphene-enhanced Raman scattering (GERS) on isotopically labelled bilayer and a single layer of pristine and partially hydrogenated graphene has been studied. The hydrogenated graphene sample showed a change in relative intensities of Raman bands of Rhodamine 6 G (R6G) with different vibrational energies deposited on a single layer and bilayer graphene. The change corresponds qualitatively to different doping of graphene in both areas. Pristine graphene sample exhibited no difference in doping nor relative intensities of R6G Raman peaks in the single layer and bilayer areas. Therefore, it was concluded that strain and strain inhomogeneities do not affect the GERS. Because of analyzing relative intensities of selected peaks of the R6G probe molecules, it is possible to obtain these results without determining the enhancement factor and without assuming homogeneous coverage of the molecules. Furthermore, we tested the approach on copper phtalocyanine molecules.

## Introduction

Enhancement of the signal in spectroscopy has crucial importance for detection and study of a low amount of species. For Raman spectroscopy, the surface-enhanced Raman scattering (SERS) technique is widely used^1^ enabling even single-molecule detection^2^. One of the restrictions of this approach is the limited stability of the metals that are needed to achieve signal enhancement^3^. Recently, it was observed that graphene itself can provide significant enhancement of the Raman signal and the so-called graphene-enhanced Raman scattering (GERS)^4–6^ was established. Note that both SERS and GERS can contribute to the molecular Raman signal together^7^. Apart from the relatively good chemical stability of graphene, it was also found that graphene quenches photoluminescence^8^, which is important for practical experiments. The GERS was observed for various molecules^9–11^ and also for other 2D materials, which were employed as active substrate^12^. Furthermore, it was shown that the enhancement can be tuned by changing the Fermi energy of graphene (modified by the electrical field effect)^13^, by substitutional doping with heteroatoms^14,15^, or by chemical functionalization^16,17^. More detailed studies demonstrated that the enhancement is also a function of the phonon energy of the specific vibration and also laser excitation energy^10^. The observed effects were rationalized by a simple theoretical approach taking into account several different resonance processes^18^.

It was already shown previously that different doping of graphene induced by functionalization leads to a change in the relative intensities of individual GERS peaks of the probe molecules^16^. The overall GERS enhancement depends on the laser excitation energy, lowest unoccupied molecular orbital (LUMO), and highest occupied molecular orbital (HOMO) energies of the probe molecules, the vibrational energy of the actual molecular Raman mode, and on the Fermi energy of graphene^18^. Specifically:

1 $$\begin{array}{c}(i)\,\hslash {\omega }_{0}={E}_{L}-{E}_{H},\,\hslash {\omega }_{0}={E}_{L}-{E}_{H}+\hslash {\omega }_{q},\\ (ii)\,{E}_{F}={E}_{H}\pm \hslash {\omega }_{q},\,{E}_{F}={E}_{L}\pm \hslash {\omega }_{q},\\ (iii)\,\hslash {\omega }_{0}={E}_{F}-{E}_{H},\,\hslash {\omega }_{0}={E}_{F}-{E}_{H}+\hslash {\omega }_{q},\\ (iv)\,\hslash {\omega }_{0}={E}_{L}-{E}_{F},\,\hslash {\omega }_{0}={E}_{L}-{E}_{F}-\hslash {\omega }_{q},\end{array}$$

where $\hslash {\omega }_{0}$ is the energy of the excitation radiation, $\hslash {\omega }_{q}$ is the energy of the molecular vibration involved, *E*_*L*_ and *E*_*H*_ are the energies of the molecular LUMO and HOMO states, respectively, and *E*_*F*_ is the Fermi energy of graphene.

For a given vibrational energy of a given molecule, measured in resonance with a given laser, the enhancement depends only on the doping level of graphene. Because of the huge photoluminescence signal of R6G molecules that are not placed on graphene, it is not possible to measure Raman spectra and therefore it is impossible to calculate the enhancement factor. However, as shown previously, even without knowledge of the enhancement factor, the effect of different doping of graphene can be rationalized by comparing relative intensities of individual GERS peaks^16^.

It was shown that the substrate significantly affects the graphene layer^19,20^. The effects of the substrate may include doping^21^, strain^22^, and/or mixing of electronic states^23^. The easiest approach to study the effect of the substrate is a comparison of the behavior of single-layer graphene and turbostratic graphene bilayer on SiO_2_/Si substrate^24^. Hence, one can compare graphene on Si/SiO_2_ vs graphene on graphene. To differentiate between the top and bottom layers of the graphene bilayer in the Raman spectra, one can employ isotopic labelling of the graphene layers: one graphene layer is prepared using natural methane gas while the other by ^13^C-enriched gas. The different atomic masses of ^13^C and ^12^C result in different Raman shifts of the Raman modes and therefore ^12^C and ^13^C graphene layer can be easily distinguished^25^.

Graphene as a monatomic layer has a potential for application in ultrasensitive sensor devices. Apart from photonic sensors employing the enhanced Raman signal^26^, sensors based on graphene field-effect transistors^27,28^ or resistivity changes^29,30^ have been widely studied. However, the main drawback of graphene sensors is their low or zero selectivity. This can be improved by the modification of graphene^31,32^. Another approach is to involve multiple detection techniques. Hydrogenated graphene has been suggested recently as a platform for electrochemical sensing^33^. Therefore, combining electrochemical sensing with GERS could extend the possibilities of hydrogenated graphene for sensor applications. Therefore, understanding the GERS effect employing hydrogenated graphene is required. Furthermore, hydrogen in hydrogenated graphene can be replaced by other functional species^34^, opening a large area of possible functionalized graphene substrates for GERS that could achieve higher enhancement for specific molecules.

In this work, we compare the GERS effect on pristine and hydrogenated graphene using a probe molecule, R6G. We also addressed the effect of the substrate by evaluation of the GERS on hydrogenated graphene on graphene and SiO_2_ substrates. We employed atomic force microscopy (AFM) and Raman spectroscopy to address the properties of the graphene, which enables us to correlate changes in graphene status with the changes of the GERS. Thanks to comparing the relative intensities of the Raman bands of R6G we were able to explain our results by theoretical predictions^18^ without determining the absolute enhancements factors and the density of the R6G molecules on individual substrates. Copper phtalocyanine molecules (CuPc) were tested on the SLG/BLG system as well.

## Results

**Figure 1:**
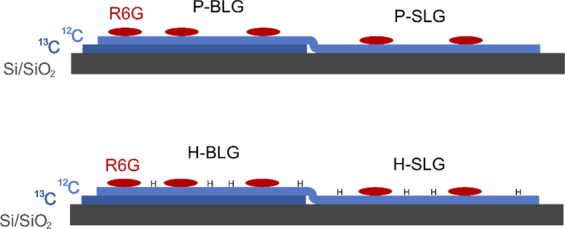
A schematic depiction of all four studied samples; pristine bilayer graphene (R6G/P-BLG), pristine single-layer graphene (R6G/P-SLG), hydrogenated bilayer graphene (R6G/H-BLG), and hydrogenated single-layer graphene (R6G/H-SLG).

The layout of the samples is shown in Fig. 1. Isotopically labelled ^13^C graphene layer partially covers Si/SiO_2_ substrate and ^12^C graphene layer is placed on top of that structure and finally, the R6G molecules are deposited. The region where the pristine ^12^C graphene layer is placed on the ^13^C graphene layer is labelled as R6G/P-BLG. The region where the pristine ^12^C graphene layer is placed directly on the Si/SiO_2_ substrate is labelled as R6G/P-SLG. In the second case, the graphene sample was hydrogenated before the deposition of R6G molecules and hydrogenated graphene bilayer region is labelled as R6G/H-BLG while hydrogenated single layer region is labelled as R6G/H-SLG.

The GERS is sensitive to the status of graphene, therefore, it is necessary to characterize graphene properties in detail. Both pristine and hydrogenated graphene samples consisted of ^12^C CVD graphene transferred over ^13^C CVD graphene. For the purpose of the measurement, we intentionally chose a spot with a crack in the bottom ^13^C graphene layer. Thus, we could study the samples with ^13^C/^12^C bilayer graphene (R6G/P-BLG, R6G/H-BLG) and ^12^C single-layer graphene (R6G/P-SLG, R6G/H-SLG) at the same time.

**Figure 2:**
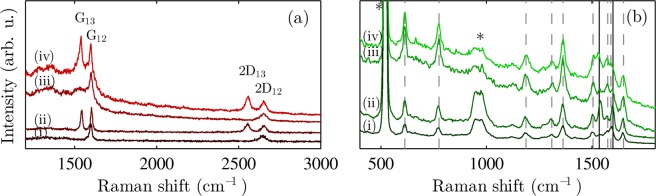
Typical Raman spectra of R6G/P-SLG (i), R6G/P-BLG (ii), R6G/H-SLG (iii), and R6G/H-BLG (iv) with 633 nm laser (**a**). The G and 2D bands originating from ^12^C and ^13^C isotopes are labelled. The GERS signal of the R6G molecules on R6G/P-SLG (i), R6G/P-BLG (ii), R6G/H-SLG (iii), and R6G/H-BLG (iv) samples measured with 532 nm laser (**b**). The R6G bands are marked by grey dashed lines, solid black lines show the position of ^13^C and ^12^C G bands. Peaks coming from Si substrate are marked by asterisk. The spectra are vertically shifted for clarity.

The samples were measured using Raman spectroscopy (Fig. 2(a)). For this experiment, we selected the excitation laser wavelength of 633 nm, which is off resonance with the R6G molecules, and therefore only graphene-related Raman modes are present in such spectrum. The most prominent Raman modes in graphene are the D, G, and 2D bands. The D mode is associated with the presence of structural defects. The G mode originates from the doubly degenerated phonon mode in the centre of the Brillouin zone, while the 2D mode originates from a second-order process involving two transverse optical phonons^35^. In the Raman signal from the isotopically labelled bilayer, the peaks coming from ^13^C graphene are shifted with respect to ^12^C graphene peaks (Fig. 2(a)) due to different atomic mass^19^.

Before hydrogenation, the Raman spectrum of graphene consisted of the G and 2D modes without any sign of the D band. The successful partial hydrogenation is demonstrated by the appearance of the D peak^36^ (Fig. 2(a)).

When measuring the samples in the same areas with a laser wavelength of 532 nm, apart from the peaks coming from graphene, R6G bands appear (Fig. 2(b)). The Raman peaks of the R6G molecules are located at around 610, 775, 1180, 1310, 1360, 1505, 1575, 1590, and 1650 cm^−1^. The photoluminescence is quenched on both pristine and hydrogenated graphene layers and the Raman peaks are visible also in both cases. These results are consistent with those observed previously for fluorinated and 4-nitrophenyl functionalized graphene^37,38^.

**Figure 3:**
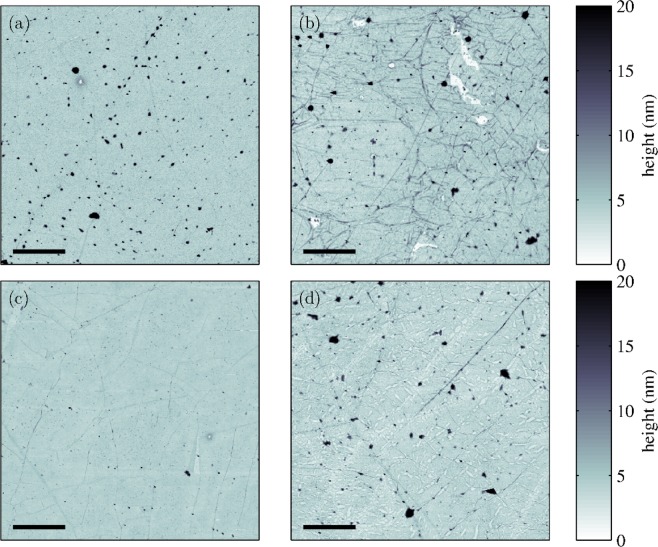
AFM topography pictures of the R6G/P-SLG (**a**), R6G/P-BLG (**b**), R6G/H-SLG (**c**), and B-SLG (**d**) samples.

Both samples with pristine and hydrogenated graphene were characterized using AFM (Fig. 3). From AFM images, no significant difference in topography between pristine and hydrogenated samples can be seen. In both cases, bilayer graphene shows more wrinkled surface, which is caused by easier relaxation of the top graphene layer^39^.

**Figure 4:**
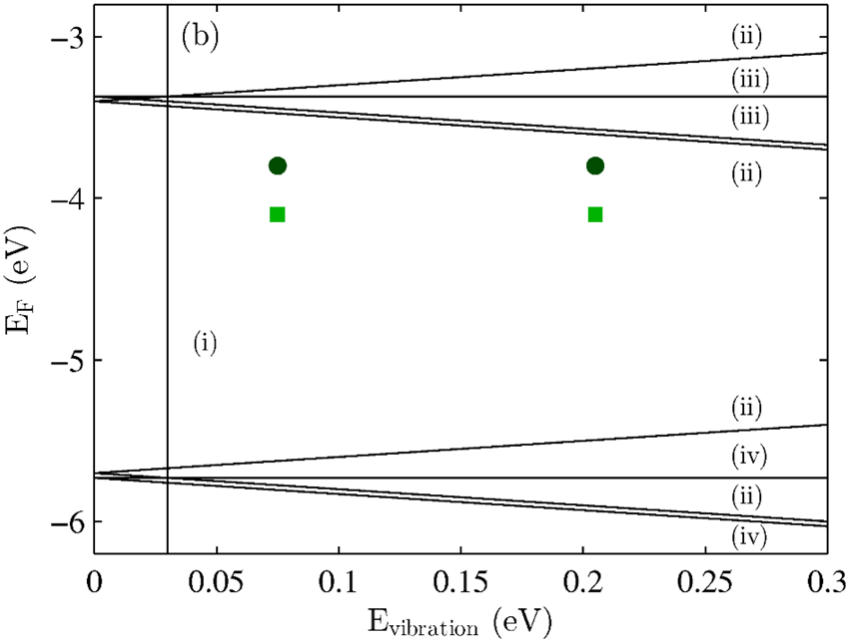
Theoretical plot of the GERS enhancement (black lines) according to Eq. 1 labelled with the number of corresponding equation. The points corresponding R6G/H-SLG (dark green circles) and R6G/H-BLG (light green squares) samples are depicted.

Because the GERS enhancement depends on the actual vibrational energy^16,18^, for the main analysis we selected two peaks with the lowest and highest vibrational energies (610 cm^−1^ and 1650 cm^−1^, 0.075 eV and 0.205 eV, respectively). The more the peaks are separated, the bigger is the difference in their enhancement. This effect is schematically shown in Fig. 4, where the black lines show the conditions for maxima of GERS enhancement (Eq. 1)^16,18^. We assumed the Fermi energy of the intrinsic graphene layer to be at −4.6 eV and the LUMO and HOMO energies of the R6G molecules to be at −3.4 eV and −5.7 eV, respectively^8^. The data points shown in Fig. 4 (corresponding to the R6G/H-SLG and R6G/H-BLG samples) were shifted because of different doping calculated from the Raman shifts of the G and 2D bands as discussed later. The dark green circles then represent R6G Raman bands at 610 cm^−1^ and 1650 cm^−1^ on R6G/H-SLG. The light green squares, corresponding to R6G/H-BLG, are plotted for lower Fermi energy of graphene because the bottom layer of hydrogenated graphene showed higher doping level than the top one. From Fig. 4, it follows that the high-vibrational-energy peak of R6G is closer to the maximum of the GERS enhancement condition and therefore with increasing p-doping of graphene, the high-energy peak will be more enhanced than the low-energy R6G Raman peak. Therefore, we can expect that the intensity of the R6G Raman band at 1650 cm^−1^ related to the intensity of the R6G band at 610 cm^−1^ will be higher for more doped graphene.

**Figure 5:**
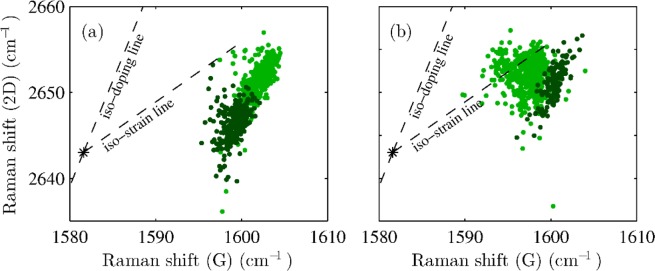
Correlation plots of the Raman shifts of the G and 2D modes of R6G/P-SLG ((**a**), dark green data points), R6G/P-BLG ((**a**), light green data points), R6G/H-SLG ((**b**), dark green data points), and R6G/H-BLG ((**b**), light green data points) samples. The iso-strain and iso-doping lines are depicted as well.

Figure 5 shows the correlation plots of the Raman shifts of the G and 2D modes, which provides information on strain and doping of graphene. The Raman maps used for the doping and strain analysis were acquired using a laser with excitation wavelength of 633 nm. Such laser energy is out of resonance with the R6G molecules, therefore their Raman bands do not overlap with graphene bands. This approach enabled us to study doping and strain of the sample, taking into account any possible effect of the molecules themselves to graphene. A method for differentiating the effect of strain and doping was introduced by Lee *et al*.^40^. When applying strain, the Raman shift of the 2D peak versus G peak is moving along the iso-doping line (slope of 2.45, the strain sensitivity is 57 cm^−1^/%^41^) from the point of neutral and unstrained graphene, while when applying doping, the shift is along the iso-strain line (slope of 0.7^42^). The G peak upsifts for both n and p-doping^43^. In real samples, both effects are present and thanks to the known values of both iso-doping and iso-strain slopes, one can decouple the contribution of strain and doping. In case of an isotopically labelled bilayer prepared by sequential transfer, the individual layers do not electronically interact, which is demonstrated by unaffected shape of the 2D band^44^. Thus, doping and strain of individual layer can be analysed independently^45^. Effect of strain and doping on Raman spectra of interacting multilayer has been investigated previously for example by Jeon *et al*.^46^ In the case of pristine graphene sample (Fig. 5(a)) the clouds of points coming from the R6G/P-SLG and R6G/P-BLG are shifted clearly only due to strain with respect to each other. Nonetheless, in the case of hydrogenated graphene sample, the cloud of points coming from R6G/H-SLG is shifted due to both strain and also due to the doping with respect to the R6G/H-BLG sample.

**Figure 6:**
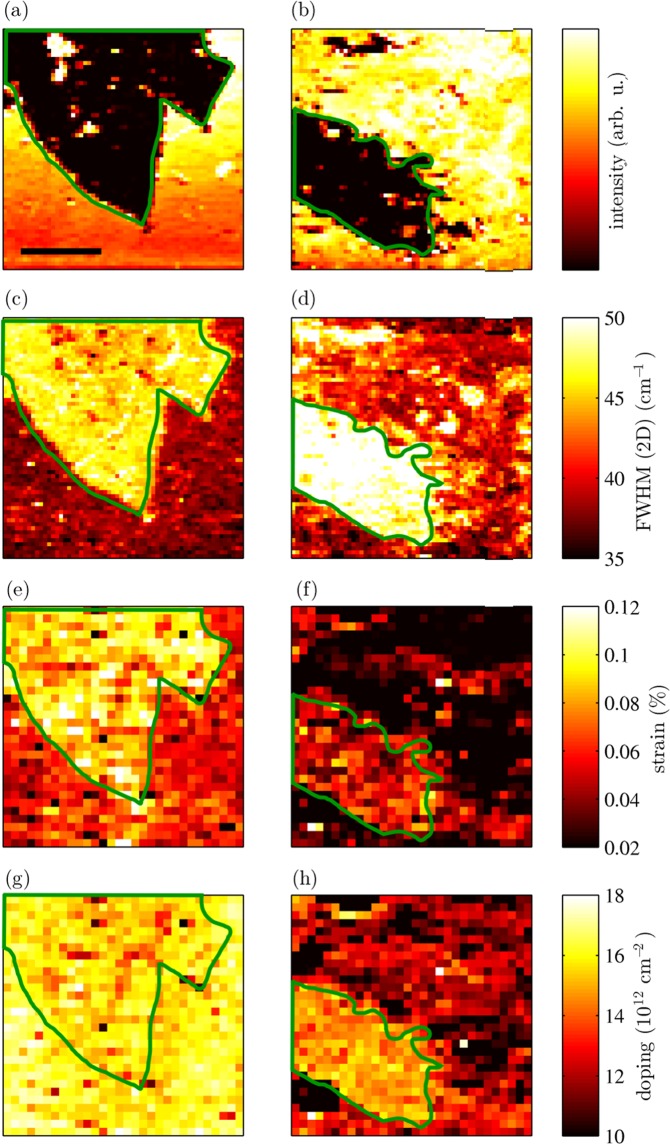
Results from the Raman mapping of pristine (left column) and hydrogenated (right column) graphene/R6G samples. Panels (a,b) show the intensity of the ^13^C 2D graphene mode of pristine and hydrogenated graphene, respectively, λ = 532 nm. Panel (c) shows FWHM of the ^12^C 2D mode of pristine graphene, panel (d) of hydrogenated graphene, λ = 532 nm. In panels (e,g) the strain and doping of ^12^C pristine graphene are displayed. Panels (f,h) display the strain and doping of ^12^C hydrogenated graphene. The measurements for strain and doping calculations were performed with an excitation laser wavelength of 633 nm and with twice as big step of mapping. The green line limits the SLG area. The length of the scale bar is 10 μm.

In Fig. 6(a,b) the map of intensities of the 2D mode of both pristine and hydrogenated ^13^C graphene clearly shows regions of single-layer ^12^C graphene and isotopically labelled graphene bilayer. These maps can be measured with a laser wavelength of 532 nm because the spectral region of the 2D mode of graphene does not overlap with the bands of the R6G molecules. Panels (c,d) of Fig. 6 show the maps of full width at half-maximum (FWHM) of the ^12^C 2D graphene mode of pristine and hydrogenated graphene, respectively. In the case of both samples, the 2D FWHM is higher when the graphene layer is directly on the Si/SiO_2_ substrate than on the other (^13^C) graphene layer. The increased width of the 2D mode indicates sub-micron strain inhomogeneities^47^. The higher 2D width of graphene on Si/SiO_2_ substrate compared with the upper layer of isotopically labelled graphene bilayer is consistent with our AFM results, showing a higher relaxation of the top layer of BLG compared to SLG sample, which is consistent with previously published measurements^48,49^.

Figure 6(e,f) shows the maps of strain in pristine and hydrogenated graphene, respectively, while panels (g,h) of Fig. 6 show the map of doping. Both strain and doping were calculated from the Raman shifts of both the G and 2D peaks (Fig. 5) as described previously^40–43,48^ and showed in Fig. 5. Because of the interaction with the substrate, p-type doping and biaxial strain were assumed. The Raman measurements for strain and doping calculations were performed with a laser using an excitation wavelength of 633 nm. For both pristine and hydrogenated graphene, the strain is smaller when ^12^C graphene layer is placed on ^13^C graphene layer than when directly placed on Si/SiO_2_ substrate. Graphene placed on another graphene layer is more likely to release strain, which is consistent with previously published data^48,49^. Since the size of the crack is larger than 10 μm, any effect on strain at the edges are expected to be negligible. Doping of the ^12^C graphene layer is higher for the R6G/H-SLG sample than with the R6G/H-BLG sample. This behaviour is consistent with published data as well^48–50^. However, in the case of pristine graphene, no significant change of doping is observed between R6G/P-SLG and R6G/P-BLG. Note that the p-doping is generally higher for pristine graphene, which is in agreement with previous findings. It was reported that partial hydrogenation induces n-doping in graphene^51^.

**Figure 7:**
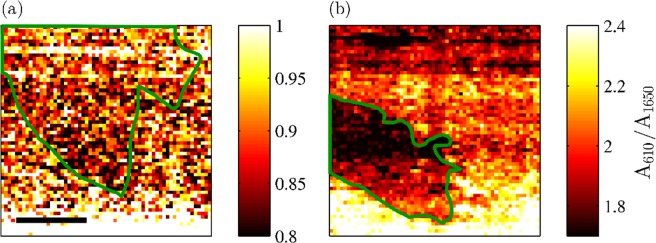
Maps of the ratios of the intensities of the R6G peaks at 610 cm^−1^ relative to the intensities of the R6G peaks at 1650 cm^−1^ of pristine (**a**) and hydrogenated (**b**) graphene samples. The marked areas indicate the single-layer graphene region. The length of the scale bar is 10 μm.

In Fig. 7 we plot the intensity of the R6G mode at 610 cm^−1^ (A_610_) relative to the intensity of the R6G peak at 1650 cm^−1^ (A_1650_) for pristine (a) and hydrogenated (b) graphene samples. One can immediately notice that while in the case of the pristine sample, the ratio (A_610_/A_1650_) is constant within the map, in the case of the hydrogenated sample the ratio (A_610_/A_1650_) is significantly lower in the R6G/H-SLG region.

As shown in Fig. 4, one can easily rationalize these findings. The only parameter of the graphene layer that varies within the maps and that influences the magnitude of the enhancement is the doping of graphene. The pristine graphene sample does not show any change in doping for single layer and bilayer regions. It also does not show any change in the relative intensity of the R6G mode at 610 cm^−1^ with respect to the R6G mode at 1650 cm^−1^. In the case of the hydrogenated graphene, one can apply a similar approach: (i) R6G Raman peaks with higher vibrational energy are more influenced by the GERS enhancement than the peaks with lower vibrational energy, (ii) more p-doped graphene layer will provide higher enhancement for the R6G Raman peaks. Therefore, higher doping of graphene provides higher sensitivity of the enhancement of the R6G Raman signal with respect to the vibrational energy of the particular Raman mode. In other words, with higher p-doping of graphene, the difference in enhancement between low-energy vibrational mode and high-energy vibrational mode will be higher than with less p-doped graphene. This is exactly what we observe in Figs. 6(h) and 7(b).

The observation that the ratio of the intensities of the R6G Raman peaks does not change for R6G/P-SLG and R6G/P-BLG areas provides us more information. This demonstrates that only doping of graphene and not a strain plays a role in GERS enhancement. For example, a single-layer graphene has a larger strain and broader 2D band (Fig. 6(c,e)). None of these parameters affects the GERS.

**Figure 8:**
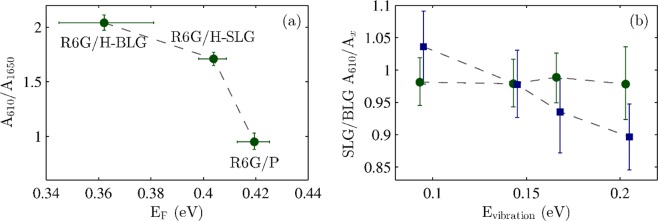
Panel (a) shows the A_610_/A_1650_ ratio plotted with respect to the doping of hydrogenated graphene bilayer (R6G/H-BLG), hydrogenated graphene single layer (R6G/H-SLG), and pristine graphene (P, R6G/P-SLG + R6G/P-BLG together). The data points are median values from the corresponding areas of the maps. The error bars represent the first and third quartiles of the datasets. Panel (b) shows relative intensities of R6G of the peak at 610 cm^−1^ and peaks at 780, 1180, 1360, and 1650 cm^−1^ (x axis), respectively, on SLG region with respect to the BLG region for pristine (green circles) and hydrogenated (blue squares) graphene samples. The data are laterally shifted for clarity.

A comparison between the doping of the graphene layers and the A_610_/A_1650_ ratios is plotted in Fig. 8(a). With increasing p-doping of graphene in the selected region, the A_610_/A_1650_ ratio is decreasing. It is worth noticing that for higher p-doping the change of the A_610_/A_1650_ ratio is faster. This is again in very good qualitative agreement with the scheme in Fig. 4 because the closer to the enhancement condition, the stronger the enhancement effect is. To verify the concept of having different enhancement for R6G bands with varied vibrational energy it is preferable to evaluate all possible R6G bands. For that we plot relative intensities of R6G of the peak at 610 cm^−1^ and peaks at 780, 1180, 1360, and 1650 cm^−1^, respectively, on SLG region with respect to the BLG region (Fig. 8(b)). Other R6G might overlap with the Raman bands of graphene. While for pristine graphene sample the ratio remains constant for all the vibrational energies, for hydrogenated graphene sample a clear decrease of the ratio with increasing vibrational energy is observed. This result perfectly match the model presented in Fig. 4.

We used the same approach for CuPc molecules. Typical Raman spectra, Raman maps of doping of the top graphene layer and of relative intensities of CuPc molecules are shown in Fig. S1. The dependence of different enhancement on SLG with respect to BLG region on molecular vibrational energy is shown in Fig. S2 together with theoretical enhancement conditions for CuPc according to Eq. 1.

## Conclusions

In this work, we studied the GERS enhancement of the R6G molecules deposited on isotopically labeled graphene bilayer and single-layer graphene to address the effects of hydrogenation and substrate. In the case of the hydrogenated graphene sample, the different doping of the single-layer (R6G/H-SLG) and bilayer graphene (R6G/H-BLG) induced different enhancements of individual R6G Raman peaks. This difference was described as a ratio of the intensities of the R6G Raman peak at 610 cm^−1^ and at 1650 cm^−1^, taking advantage of the difference in their phonon energies. This peak intensity ratio was lower in the single-layer area, which was qualitatively explained by the different doping of the single-layer and bilayer graphene. As we compared relative intensities of two R6G peaks, the value of enhancement factor was not needed and, furthermore, no assumption on the homogeneity of the coverage with R6G molecules had to be considered. The pristine sample did not show any difference in doping of the single-layer (R6G/P-SLG) and bilayer graphene (R6G/P-BLG) and consequently, it also did not show any difference in the ratio of the R6G peaks. Because of the constant ratio of the R6G peaks, we can exclude the effect of strain and strain inhomogeneity on the GERS effect because the strain differed from single layer to bilayer graphene. The relative intensities of the R6G peaks are in agreement with the doping variations of graphene even when comparing pristine and hydrogenated graphene. Analysis of the relative intensities of all accessible R6G bands on SLG and BLG region of pristine and hydrogenated graphene confirmed the theoretical concept as well. Furthermore, we also probed CuPc molecule (SI). The change of relative intensities of CuPc Raman modes agrees with the theoretical expectations and the results with R6G, however, it is not so pronounced. Therefore we analyzed relative intensities of several peaks with respect to SLG and BLG part which confirmed the observed trend.

## Experimental Methods

Graphene was prepared by a chemical vapor deposition (CVD) method on copper foil^52^. Isotopically labelled graphene layer was prepared using ^13^CH_4_ as a precursor^19^. ^13^C graphene was transferred onto the Si substrate with a 300 nm thick SiO_2_ layer (Si/SiO_2_) using the nitrocellulose-assisted method^53^. Isotopically labelled graphene bilayer was prepared by subsequent transfer of ^12^C graphene using the same method. Isotopic labelling was used to be able to study individual layers independently using Raman spectroscopy. One of the samples was hydrogenated in a high-pressure autoclave as described elsewhere^34^. Briefly, the autoclave was flushed several times with hydrogen to remove air. Then, the autoclave was filled with hydrogen at a pressure of 5 bar. The temperature was increased from room temperature to 200 °C. The reaction was carried out for 2 hours at roughly 8 bar.

The R6G molecules (Sigma-Aldrich, R4127, CAS number: 989-38-8) were deposited onto graphene by soaking the substrate in a 10^−6^ mol·l^−1^ aqueous solution for 2 minutes. The substrates were subsequently immersed in deionized water for 30 minutes to remove possible excess of the R6G molecules.

The Raman maps were measured with a Witec alpha300 R spectrometer equipped with a piezo stage. The excitation laser wavelengths of 532 nm and 633 nm with the laser power below 1 mW were used. The laser was focused on the sample with a 100× objective to a spot with a diameter of around 500 nm. All the Raman peaks were fitted using pseudo-Voigt functions.

## Supplementary information

Supplementary Information.
