# Supplementary material for: Graphene-enhanced Raman scattering on single layer and bilayers of pristine and hydrogenated graphene
Source: Sci Rep. 2020 Mar 11;10:4516. doi: 10.1038/s41598-020-60857-y (PMC7066185; doi:10.1038/s41598-020-60857-y)
Supplement: Supplementary file 1 — Supplementary Information. [file 41598_2020_60857_MOESM1_ESM.docx]

**Supplementary information**

Graphene-enhanced Raman scattering on single layer and bilayers of pristine and hydrogenated graphene

*Václav Valeš, Karolina Drogowska-Horná, Valentino L. P. Guerra, Martin Kalbáč**

J. Heyrovský Institute of Physical Chemistry, ASCR, v.v.i., Dolejškova 3, 182 23 Praha, Czechia

In order to support our results, we focused on CuPc molecules deposited on a substrate that consisted of hydrogenated SLG and BLG. The CuPc molecules were deposited onto hydrogenated SLG/BLG by soaking in a 10^–5^ mol·l^–1^ solution in methanol for 2 minutes and washed in methanol afterwards. Typical spectra for the GERS signal both from SLG and BLG measured with a laser with excitation wavelength of 633 nm are shown in Figure S1(a). While it can be seen that there is a difference in doping of the top graphene layer in SLG and BLG region (Figure S1(b)), the ratio of CuPc GERS peaks (A_750_/A_1450_) shows only slight difference (Figure S1(c)). In order to clarify the slight difference in relative intensities between SLG and BLG region we focused on relative intensities of A_750_ peak with other peaks 1145, 1310, 1345, and 1450 cm^-1^ (Figure S2 (a)). It can be seen from that graph that with increasing difference of vibrational energy between the two peaks, the difference between SLG and BLG region is becoming more significant. The change is not so significant as for R6G molecules. The main reason is that in case of CuPc the accessible range of vibrational energies (0.04 eV) is not so broad as for R6G (0.13 eV). The theoretical conditions for enhancement calculated using Equation 1 assuming the LUMO and HOMO energies of -3.5 and -5.2 eV,^1^ respectively (Figure S2(b)) show that the qualitative behaviour of the enhancement of individual peaks should be the same as for R6G. Therefore, the measured relative intensities of CuPc molecules further confirm our results.


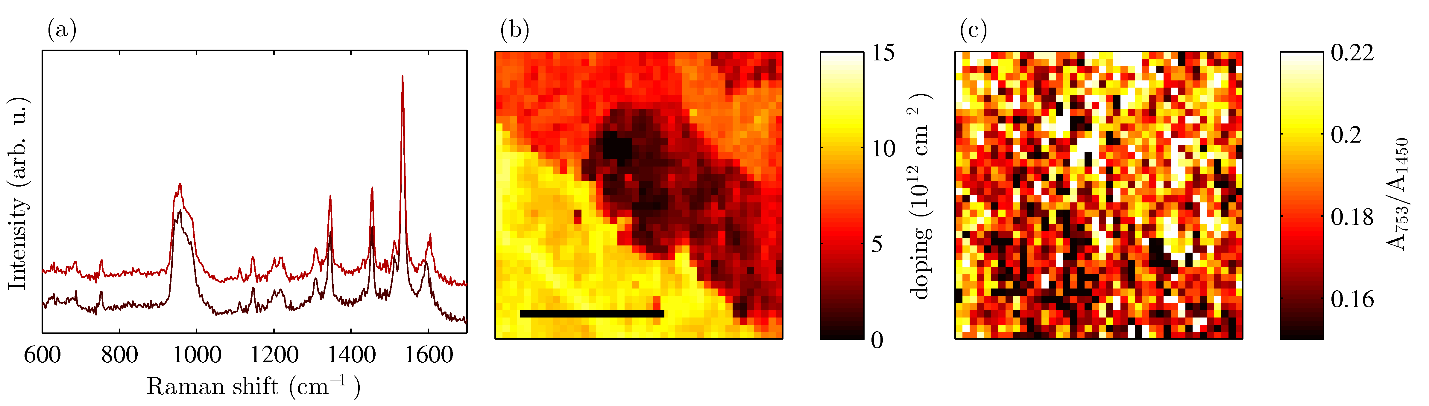


Figure S1: GERS signal of CuPc molecules on BLG (dark red line) and SLG (light red line) is plotted in panel (a). Panel (b) shows the spatial distribution of doping of the top graphene layer. The length of the scalebar is 10 μm. In panel (c) a map of the ratio of intensities (A_750_/A_1450_) of CuPc GERS signal is plotted.


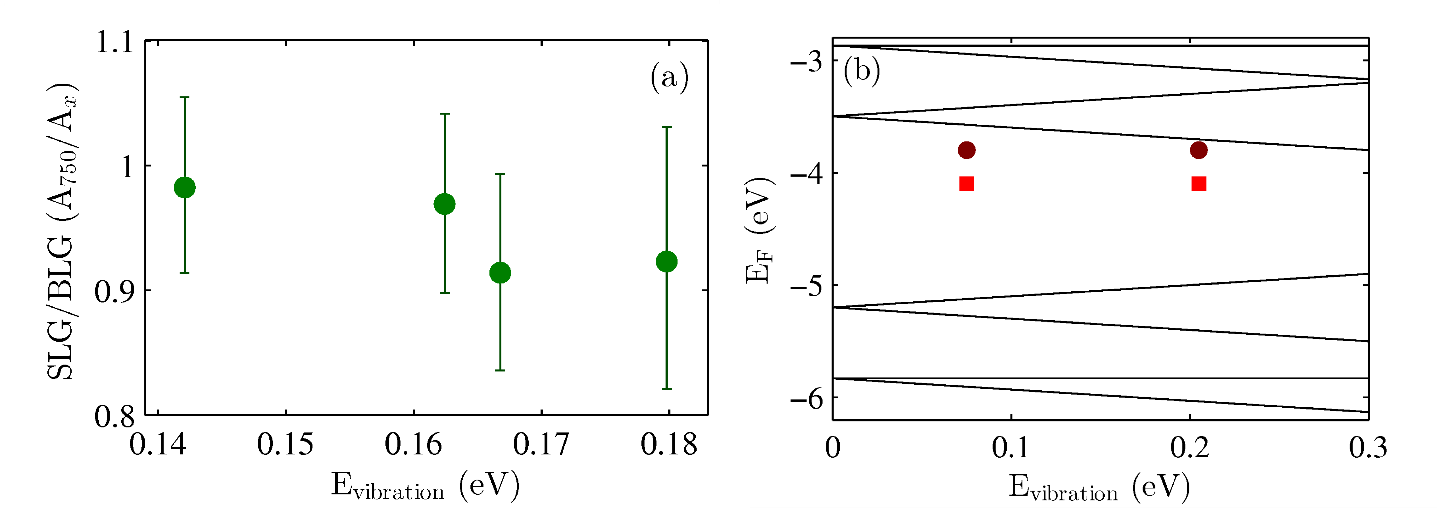


Figure S2: Panel (a) shows relative intensities of CuPc of the peak at 750 cm^-1^ and peaks at 1145, 1310, 1345, and 1450 cm^-1^ (x axis), respectively, on SLG region with respect to the BLG region. The error bars represent the first and the third quartiles of the datasets. The theoretical enhancement conditions calculated by Equation 1 for CuPc molecules are plotted in panel (b).

1. Huang, S. *et al.* Molecular Selectivity of Graphene-Enhanced Raman Scattering. *Nano Lett.* **15**, 2892–2901 (2015).
